# Genetic testing involving 100 common mutations for antenatal diagnosis of hereditary hearing loss in Chongqing, China

**DOI:** 10.1097/MD.0000000000025647

**Published:** 2021-04-30

**Authors:** Hua Hu, Peng Zhou, Jiayan Wu, Wei Lei, Yang Wang, Ying Yang, Hailiang Liu

**Affiliations:** a Second Affiliated Hospital, Army Military Medical University, Chongqing; b CapitalBio Genomics Co., Ltd., Dongguan, China.

**Keywords:** genetic testing, *GJB2*, *GJB3*, hearing loss, mitochondrial DNA, *SLC26A4*

## Abstract

To understand the possible carrier status of genes associated with hereditary hearing loss (HHL) in the general population among local residents and to give genetic counseling for pregnant women.

A total of 3541 subjects were recruited. We used multiplex PCR technology combined with next-generation sequencing technology to detect 100 hotspot mutations in 18 common deafness-related genes. The homozygous mutation screening results were verified using Sanger sequencing.

Of the 3541 participants, 37 alleles of 8 deafness genes were detected. A total of 145 (4.09%) were found to be *GJB2* gene mutation carriers, and the hotspot mutation was c.235delC (1.54%). Twenty three (0.65%) were found to be *GJB3* gene mutation carriers. A total of 132 (3.37%) were found to be *SLC26A4* gene mutation carriers, and the hotspot mutation was c.919-2A > G (0.49%). Forty four (1.24%) were found to be mitochondrial DNA mutation carriers. Sanger sequencing results verified that 2 cases were homozygous for the c.235delC mutation and that 1 case was homozygous for the c.754T > C mutation.

Genetic testing for pregnant women and their partners allows early identification of the molecular etiology of hearing loss (HL). On the one hand, it could give genetic counseling for pregnant women, such as early diagnosis of delayed deafness and drug-susceptible deafness. On the other hand, it could be used to assess hearing conditions during pregnancy, leading to prevention and timely intervention for newborns.

## 1 Introduction

Worldwide reporting of hearing loss has shown that it affects at least 30% of the population at some time in their lives, and the prevalence of moderate and severe bilateral hearing deficits (>40 dB) is 1 to 3 per 1000 live births in the healthy baby nursery population.^[1,2]^ In China, approximately 30,000 infants are born with congenital hearing loss every year.^[3]^ If hearing loss is not detected and intervened in time, infants with profound hearing loss (≥90 dB HL) will suffer permanent hearing impairment, resulting in deficits in linguistic development, cognitive abilities, and academic achievement.^[4,5]^ Approximately 50%^[6,7]^ of hearing loss cases have a genetic etiology, and more than 80 deafness genes, with more than 1200 mutations, have been verified to be associated with deafness (http://deafnessvariationdatabase.org/). Many studies of deafness molecular epidemiology in China have shown that a number of non-syndromic hearing loss (NSHL) genes are caused by only several mutated genes, such as the gap junction protein beta-2 gene (*GJB2* gene), beta-3 gene (*GJB3* gene), and *SLC26A4* gene (PDS gene), and mitochondrial DNA (mtDNA).^[8]^ Hearing loss gene identification has effectively improved the clinical diagnosis and management of deaf and hard-of-hearing people.^[9]^

Deafness is common to both men and women, and the risk of disease in offspring is significantly increased if the couple has the same deafness mutation site.^[10]^ Given that the accuracy of the postnatal hearing test is low, progressive and drug-sensitive deafness cannot be screened at times. It may assist in preventing the birth of children with hereditary hearing loss (HHL) if the deafness mutation site is confirmed in high-risk deaf couples or couples who have given birth to children with deafness.^[11]^ Therefore, genetic diagnosis and proper intervention are quite important to alleviate such problems.

There are 1.383 billion people in China, which has a great effect on genetic counseling, as a national deafness database and that provides effective molecular diagnostic data for deafness has been constructed. In recent years, genetic analysis of deaf patients has been carried out in most areas of China,^[12]^ and we also launched a nationwide genetic testing for neonatal deafness in China.^[13,14]^ However, investigations of HHL gene mutations in Chongqing are still rare.^[12,15]^

Currently, it is thought that DNA detection is the “gold standard” for molecular diagnosis.^[16]^ The common mutation in deafness genes is mainly the single-gene biallelic mutation,^[17]^ and many mutational hot spots from many deafness genes have been revealed. However, there still exist some limitations:

1. only a few genes are routinely screened,
2. most deafness genes are ignored, and
3. the carrier frequency of some alleles is still unknown.

In this study, the latest high-throughput sequencing technique can sequence millions of DNA molecules in parallel simultaneously, and we used this technique to screen the 100 common mutations in 18 deafness-associated genes in 3380 pregnant women, which provided a more accurate estimation of deafness-associated gene mutations and further guidance for its positive intervention and cure.^[17]^

## 2 Materials and methods

### 2.1 Enrollment and ethics statement

From May 2017 to February 2018, 3380 pregnant women underwent deafness genetic testing and reported 342 deafness mutation cases (see supplementary material Table S1), including 2 cases of heterozygous *GJB2* with heterozygous SLC26A4 and 1 case of heterozygous *GJB2* with a mtDNA mutation. To assess the genotype of fetal deafness, we communicated with these 342 pregnant women and their partners, and the 161 husbands agreed to undergo genetic testing. All subjects were recruited from the Second Affiliated Hospital of Army Military Medical University. The pregnant women recruited in this study were pregnant women who visited our hospital for routine prenatal checkup. The majority of these pregnant women had normal hearing, but 3 cases involved hearing impairment. All the subjects gave written, informed consent to participate in this study. This study was approved by the Ethics Committee of the Second Affiliated Hospital of Army Military Medical University.

### 2.2 Variant analysis

Genomic DNA was extracted from 3 to 5 ml of peripheral blood samples from the subjects using a blood DNA kit (Tiangen Biotech, Beijing, China) following the manufacturer's protocol. Briefly, 20 μl of proteinase K was added to a peripheral blood sample for DNA extraction, and a NanoDrop 8000 ultraviolet-visible spectrophotometer (Thermo Fisher Scientific, DE, USA) was used to determine the quality of DNA. Then, library construction, quality control, and sequencing template preparation were performed according to the instructions of a Blood DNA LQ kit. For DNA sequencing, the JingXin BioelectronSeq 4000 System (CFDA registration permit No. 20153400309) semiconductor sequencer was used. A deafness diagnostic screening panel developed by our laboratory group was used for gene mutation screening. One hundred common mutational alleles and their neighboring sequence regions of 18 deafness-associated genes, including *GJB2*, *SLC26A4*, and *GJB3*, as well as mtDNA (see supplementary material Table S2), were detected by using a deafness diagnostic screening panel (CapitalBio Genomics Co., Ltd., China). Those 100 common mutations include a high-frequency mutation site in the Chinese population. Multiplex polymerase chain reaction (PCR) technology was used to build the library, and Ion Torrent^TM^ next-generation sequencing (NGS) technology was used to explore the mutation alleles in the patients. If the mutation frequency was 0, it was considered wild type; if the mutation frequency was 0.5, it was heterozygous; if the mutation frequency was 1, it was homozygous. The pathogenicity of the variant was determined according to American College of Medical Genetics and Genomics (ACMG) guidelines.

### 2.3 Sanger DNA sequencing

Homozygous carriers that were detected by multiplex PCR technology combined with next-generation sequencing technology were confirmed via Sanger sequencing. Primer 3 (v. 0.4.0; http://primer3.ut.ee) was used for primer design. PCR was performed with 50 ng of genomic DNA and Taq DNA polymerase (Sigma, St. Louis, USA) using standard protocols. PCR was performed in a 50 ml reaction mixture. An ABI 3100 (ABI, Foster City, CA, USA) was used for sequencing and data collection. DNA sequence analysis was performed with DNASTAR Lasergene software.

### 2.4 Statistical analysis

The hearing genetic testing data for 3380 pregnant women and 161 of their partners were analyzed, a statistical analysis was conducted. The carrier frequency of each mutated gene was calculated by the percentage of subjects with the gene mutation among the total samples (each gene was counted separately; that is, 1 pregnant woman carrying 2 gene mutations was counted twice). The formula for the allele frequency of each mutation was as follows: allele frequency = (homozygous cases∗2+heterozygous cases)/ (total cases∗2)∗100%.

## 3 Results

**Figure 1:**
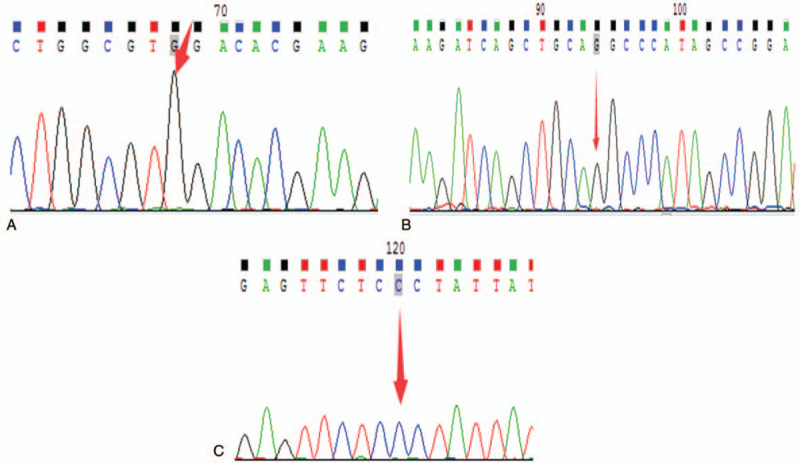
Sanger sequencing results verifying the homozygous variant. (A) Patient A carried a homozygous *GJB2* c.235delC mutation. (B) Patient B carried a homozygous *GJB2* c.235delC mutation. (C) Patient C carried a homozygous *SLC26A4* c.754T > C mutation.

A total of 3380 pregnant women and 161 of their partners were subjected to genetic testing for 100 common mutations in 18 deafness-related genes, and a statistical analysis was conducted. The carrier frequency for the genes detected is shown in Table 1. The allele frequency of each common mutation is presented in Table 2; analysis was performed in combination with the DVD (Deafness Variation Database; http://deafnessvariationdatabase.org/) and ClinVar database of NCBI (https://www.ncbi.nlm.nih.gov/clinvar/) to obtain more allele information. Five couples with positive screening results are presented in Table 3. The sequences from pregnant women with homozygous mutations, which were detected by multiplex PCR technology combined with next-generation sequencing technology, were verified by Sanger sequencing, as shown in Figure 1. In total, 37 alleles of 8 deafness genes, *GJB2*, *SLC26A4*, *GJB3*, *TMC1*, *MT-RNR1*, *MT-CO1*, *MT-TH*, and *MT-TL1*, were detected in the 3380 pregnant women.

**Table 1:** Carrier frequency of 100 hotspot mutations in 3380 pregnant women and 161 husbands.

Genetic tests	Case counts	Carrier frequency (%)
GJB2	145	4.09%
GJB3	23	0.65%
SLC26A4	132	3.73%
TMC1	1	0.03%
mtDNA	44	1.24%
Total	345	9.74%

**Table 2:** Variations detected out in 3380 pregnant women and 161 husbands.

Gene	cDNA change	Amino acid change	Consequence	Category	Mode of inheritance	Homo	Hetero	Allele frequency (%)^∗^
GJB2	c.235delC	Frameshift	Deletion	Pathogenic	AR	1	107	1.54%
	c.299-300delAT	Frameshift	Deletion	Pathogenic	AR	0	24	0.34%
	c.416G>A	p.Ser139Asn	Missense	Pathogenic	AR	0	7	0.10%
	c.257C>G	p.Thr86Arg	Missense	Pathogenic	AR	0	1	0.01%
	c.512insAACG	Frameshift	Insertion	Pathogenic	AR	0	1	0.01%
	c.176–191del16	p.Gly59Alafs	Deletion	Pathogenic	AR	0	4	0.06%
GJB3	c.538C > T	p.Arg180Ter	Nonsense	Unknown significance	AD	0	7	0.10%
	c.547G > A	p.Glu183Lys	Missense	Unknown significance	AD	0	12	0.17%
	c.423delATT	p.Ile141del	Deletion	Pathogenic	AR	0	1	0.01%
	c.497A > G	p.Asn166Ser	missense	Pathogenic	AR	0	3	0.04%
SLC26A4	c.919–2A > G	Aberrant splicing	Missense	Pathogenic	AR	0	35	0.49%
	c.919–18T > G	/	Missense	Benign	AR	0	32	0.45%
	c.1594A > C	p.Ser532Arg	Deletion	Pathogenic	AR	0	2	0.03%
	c.281C > T	p.Thr94Ile	Deletion	Pathogenic	AR	0	1	0.01%
	c.920C > T	p.Thr307Met	Deletion	Pathogenic	AR	0	1	0.01%
	c.2168A > G	p.His723Arg	Missense	Pathogenic	AR	0	2	0.03%
	IVS16–6G > A	Aberrant splicing	Missense	Pathogenic	AR	0	16	0.23%
	c.754T > C	p.Ser252Pro	Missense	Pathogenic	AR	1	1	0.04%
	c.1975G > C	p.Val659Leu	Missense	Likely pathogenic	AR	0	4	0.06%
	c.589G > A	p.Gly197Arg	Missense	Likely pathogenic	AR	0	1	0.01%
	c.697G > C	p.Val233Leu	Missense	Pathogenic	AR	0	16	0.23%
	c.259G > T	p.Asp87Tyr	Missense	Pathogenic	AR	0	1	0.01%
	c.1079C > T	p.Ala360Val	Missense	Pathogenic	AR	0	1	0.01%
	c.1174A > T	p.Asn392Tyr	Missense	Pathogenic	AR	0	4	0.06%
	c.1226G > A	p.Arg409His	Missense	Pathogenic	AR	0	2	0.03%
	c.1343C > T	p.Ser448Leu	Missense	Pathogenic	AR	0	2	0.03%
	c.1693insA	Aberrant splicing	Missense	Pathogenic	AR	0	1	0.01%
	c.2027T > A	p.Leu676Gln	Missense	Pathogenic	AR	0	1	0.01%
	c.1229C > T	p.Thr410Met	Missense	Pathogenic	AR	0	6	0.08%
	c.812A > G	p.Asp271Gly	Missense	Pathogenic	AR	0	1	0.01%
	IVS14+1G > A	Aberrant splicing	Missense	Pathogenic	AR	0	1	0.01%
TMC1	c.150delT	Frameshift	Deletion	Pathogenic	AR	0	1	0.01%
MT-RNR1	m.1494C > T	*12SrRNA*	Missense	Pathogenic	MI	1	0	0.03%
	m.1555A > G	*12SrRNA*	Missense	Pathogenic	MI	11	3	0.35%
MT-TH	m.12201T > C	*tRNA-HIS*	Aberrant splicing	Pathogenic	MI	0	1	0.01%
MT-TL1	m.3243A > G	*tRNA-LEU*	Aberrant splicing	Pathogenic	MI	0	1	0.01%
MT-CO1	m.7444G > A	*tRNA*^Ser(UCN)^	Missense	Pathogenic	MI	27	0	0.76%

**Table 3:** The genotypes of 5 couple carried deaf genes mutation.

	Genotype of the women		Genotype of the husband		
Family	Gene	Nucleotide change	Gene	Nucleotide change	Newborn follow-up
1	*GJB2*	Heterozygous c.235delC	*SLC26A4*	Heterozygous c.919–18T > G	Normal hearing
2	*GJB2*	Heterozygous c.299–300delAT	*GJB2*	Heterozygous c.235delC	Normal hearing
3	*SLC26A4*	Heterozygous c.1174A > T	*GJB2*	Heterozygous c.416G > A	Normal hearing
4	*GJB3*	Heterozygous c.423de1ATT	*MT-RNRI*	Heterozygous m.1555A > G	Normal hearing
5	*GJB2*	Heterozygous c.299–300delAT	*SLC26A4*	Heterozygous c.697G > C	Normal hearing

### 3.1 GJB2

A total of 4.09% pregnant women (145/3541) carried the *GJB2* gene mutation, which included mutations at 6 sites (c.235delC, c.299_300delAT, c.416G > A, c.257C > G, c.512insAACG, and c.176–191dell) (Table 2**)**. In 66 pregnant women with the c.235delC mutation, 2 of them were homozygous for the c.235delC mutation, and both mutations were verified by Sanger sequencing (Fig. 1). There were 11 *GJB2* compound heterozygous mutations. In addition, heterozygous c.235delC and c.416G > A mutations were detected in 2 partners of the pregnant women with positive gene mutations (Table 3).

### 3.2 SLC26A4

The *SLC26A4* gene mutation carrier frequency was 3.73% (132/3541) in this study (Table 1). A total of 17 mutation alleles were detected (Table 2), 1 pregnant woman carried a homozygous c.754T > C mutation, and the screening results were verified by Sanger sequencing (Fig. 1). The variant c.919–2A > G in *SLC26A4* had the highest allele frequency. In addition, heterozygous c.919–18T > G and c.697G > C mutations were detected in 2 partners of the pregnant women with positive gene mutations (Table 3).

### 3.3 GJB3

For the *GJB3* gene, the mutation carrier frequency was 0.65% (23/3541) (Table 1), and 3 mutation alleles were detected in this study (Table 2). Among them, c.423delATT and c.497A > G were pathogenic with autosomal recessive inheritance mode, whereas c.538C > T and c.547G > A were described as having unknown significance, with an autosomal dominant mode of inheritance, according to the ClinVar and DVDs.

### 3.4 Mitochondrial DNA (mtDNA)

For mtDNA mutations, approximately 44 pregnant women were showed mutations, including m.1494C > T and m.1555A > G in the *MT-RNR1* gene, m.7444G > A in the *MT-CO1* gene, m.12201T > C in the *MT-TH* gene and m.3243A > G in the *MT-TL1* gene (Table 2). Thirty nine pregnant women with mitochondrial homoplasmic mutations, including 27 m.7444G > A in the *MT-CO1* homoplasmic mutation, 11 m.1555A > G in the *MT-RNR1* homoplasmic mutation and 1 m.1494C > T in the *MT-RNR1* homoplasmic mutation. One partner of a pregnant woman had a heteroplasmic m.1555A > G mutation (Table 3).

### 3.5 Other genes

Except for the genes mentioned above, a pregnant woman who carried the heterozygous *TMC1* c.150delT mutation was also found. In addition, there were no other gene mutations found, which suggested that the genes above (*GJB2*, *GJB3*, *SLC26A4*, *TMC1*, *MT-RNR1*, *MT-TH*, *MT-TL1*, *MT-CO1*) contained the main mutations carried by common neonates in the Chongqing area.

## 4 Discussion

We used a panel that included 100 common mutations in 18 deafness-related genes and that was seldom used in other hearing genetic testing studies. The panel used in this study involved multiple-PCR combined with Ion Torrent next-generation sequencing technology, and it covered 100 hot spot alleles in 18 common deafness genes, which encompass high-frequency mutation sites in the Chinese population. Our previous study showed that a significant difference (*P* < .001) existed between the audio-no-pass group and the randomly selected group revealed by this panel, which resulted in a high precision of sensitivity and reliability in a large sample study.^[17]^

An epidemiological survey in China found that the prevalence rate of congenital deafness is increasing annually.^[16]^ China is a large country, with 1.3 billion people of 56 ethnicities. For effective genetic testing and accurate counseling, comprehensive genetic analysis of deaf patients in most Chinese regions has been performed. Data released by the Yunnan Province Disabled Persons Federation showed that 7.47% (477/6383) of handicapped out-of-school children experienced hearing impairment. A previous study via microarray chip inspection reported that the carrier frequency of Xuzhou was 4.45%.^[11]^ Moreover, Dai's paper reported that the carrier frequency of Beijing was 4.508%.^[14]^ A study including 17,000 Chinese newborns from Chengdu reported that the carrier frequency of Chengdu was 3.08%.^[18]^ However, little is known about Chongqing, which is located in the Southeastern region of mainland China. We discovered that the positive mutation carrier frequency of HHL was 9.74% in the Chongqing area through a large screening sample in this study. This population is a comparatively large population; thus, the figure could be reliable and representative, and it is important to screen and genetically counsel people with hearing loss in the Chongqing area. Thus, the goal of this genetic testing is not to understand the possible carrier status of genes associated with HHL in the general population among local residents but rather to collect information for genetic counseling of pregnant women, for early diagnosis of delayed deafness, and for drug-susceptible deafness.

Genetic mutations related to hearing loss in the Chinese population have been studied for many years. Previous studies have shown that *GJB2* was the first disease-causing gene identified for Chinese non-syndromic hearing loss,^[19]^ and *GJB2* mutations are the most common causes of deafness in China.^[20]^ Similarly, mutations in *GJB2* were the most common mutations responsible in the present study. The carrier frequency of *GJB2* was 4.09% in this study, which is consistent with the findings of previous reports.^[14]^ Mutation of c.235delC has also been reported to be prevalent in Asia. *GJB2* 235 del C was also observed to be the most common deafness-associated mutation in the Yongchuan district of the Chongqing area in a previous study,^[21]^ and it was also found to have a high allele frequency in our study. The frequency of c.235delC was 1.54% in this study, which falls within the reported range of 0.8% to 1.96% among East Asian populations.^[5,22–24]^ Mutation of c.235delC was the most prevalent mutation in the Chongqing area, which was consistent with findings in most areas of China.^[25–27]^ In the present study, 1 pregnant woman carried a heterozygous c.235delC mutation in *GJB2*, and her husband carried a heterozygous c.919–18T > G mutation in *SLC26A4*. Follow-up of their newborn baby revealed that normal hearing. In addition, another woman carried a homozygous c.235delC mutation in this study. She had hearing loss and profound hearing impairment in the left ear and moderately severe hearing impairment in the right ear.

Genetic analysis of patients with large vestibular aqueducts in China showed that at least 1 *SLC26A4* (pendrin) gene mutation was found in 95% to 97% of these patients, confirming that large vestibular aqueduct syndrome is a genetic disease with specific mutations in China.^[28]^ However, a multi-institutional study of large vestibular aqueduct patients in the United States and England showed that only 27% of these patients had an *SLC26A4* gene mutation.^[29]^ Thus, the mutation hotspots of *SLC26A4* differ between populations in different nations and areas. In this study, the carrier frequency of *SLC26A4* was 3.73%, which was similar to the results in Zhao's report.^[30]^ A total of 21 mutation alleles were detected, including 1 homozygous c.754T > C mutation. The hotspot mutation of *SLC26A4* was c.919–2A > G, with an allele frequency of 0.49%, followed by c.919-18T > G, with an allele frequency of 0.45%, which is slightly lower than the findings in Zhao's report.^[30]^*SLC26A4* governs Pendred syndrome, which is one of the most common forms of syndromic hearing Loss. Pendred syndrome may occur at any age from birth to adolescence and is associated with cold, fever, mild craniocerebral trauma, barotrauma, or other causes of increased intracranial pressure.^[31]^ One subject with a homozygous mutation in c.754T > C was deaf.

The carrier frequency of mitochondria DNA in the 3380 pregnant women and 161 of their partners was 1.24% in this study. Once a mitochondrial DNA mutation was identified, the carrier must prevent drug-induced deafness by avoiding the use of aminoglycoside antibiotics over time.^[32,33]^ In our study, we found that pregnant women with normal hearing carried mitochondrial gene mutations. Because the mitochondrial gene is maternal, the children would not be predicted to have HHL if their father carried mitochondrial gene mutations. Even though the mother had HHL caused by the mitochondrial gene mutation, if their children did not use ototoxic drugs while growing up, the children could be carriers with normal hearing.^[34]^ Therefore, we advised carriers of mitochondrial DNA mutations to be aware of hearing susceptibility very early in life and to pay attention to the health of their hearing. Our research also provides important information for medical practitioners to make decisions and helps to improve control measures.

The mitochondrial *12S rRNA* gene mutation is associated with maternal inheritance, and the application of aminoglycosides resulted in irreversible hearing loss.^[35]^ In familial cases of ototoxicity, aminoglycoside hypersensitivity is often maternally transmitted. In the human mtDNA genome, m.1555A > G is the most common mutation in this gene. The *12S rRNA* gene has been proposed to be the primary targeting site for aminoglycosides. The identified non-syndromic deafness-causing mtDNA mutations include m.1555A > G,  m.1494C > T, and m.1095 T > C and mutations at position 961 in the *12S rRNA* gene.^[36–38]^ Currently, it is estimated that these mutations are present in approximately 3.10% of patients with NSHL. In the present study, 3380 pregnant women and 161 of their partners were screened for the mtDNA *12S rRNA* gene by NGS. We detected 44 variants in *12S rRNA*. The detection rate of mtDNA *12S rRNA* in this study was 1.24%, which is higher than that in a previous study (the MTRNR1 pathogenic mutation carrier rate was 0.4%).^[39]^ Screening the mtDNA *12S rRNA* gene by NGS may aid in effectively identifying a large number of individuals who have this gene mutation, are sensitivity to aminoglycoside drugs, have normal hearing abilities and inherited the mutation maternally. Thus, through education and application of medication, deafness in high-risk groups may be avoided. In addition, scientific genetic counseling, pre- natal diagnosis and intervention may be performed over several generations, thus preventing the passing of this gene mutation in the family.

In this study, a total of 342 pregnant women were detected as carriers of deafness, and their partners were advised to be tested for possible carrier status. Of the 342 partners, 161 were tested, and 5 were found to carry a different type of deaf gene mutation (Table 3); the participation rate was 46.53%. Xiaoli et al^[40]^ conducted a survey: the results showed that, assuming that there is a deafness gene mutation in their partner, 86.9% of interviewees were willing to undergo relevant examinations and diagnoses. In their study, women were more involved than men and paid more attention to the genes they carried. This would be an important step towards establishing a deafness database and ethical rules, which could provide an accurate and reliable genetic counseling system for people with deafness.^[41]^ A total of 342 pregnant women were detected as carriers of deafness, and we conducted telephone follow-ups on the hearing ability of the newborn babies of all the pregnant women. Among the 214 people reached by phone, 212 had infants with normal hearing, and 2 had undergone induced labor due to abnormal growth. Through this study, we believe that it is necessary for the general public to familiarize themselves with knowledge concerning hereditary deafness. Unlike conventional newborn hearing screening, genetic screens for pregnant women and their partners allow early identification of the molecular etiology of HL. On the one hand, the information could be used to give genetic counseling for pregnant women, such as early diagnosis of delayed deafness and drug-susceptible deafness. On the other hand, the information could be used to assess hearing condition during pregnancy, leading to prevention and timely intervention for newborns.

## 5 Conclusion

We discovered that the positive mutation carrier frequency of hereditary hearing loss was 9.74% in the Chongqing area through a large screening sample. This population is a comparatively large population; thus, the figure could be reliable and representative, and it is important to screen and genetically counsel people with hearing loss in the Chongqing area. Genetic screens for pregnant women and their partners allow early identification of the molecular etiology of hearing loss. On the one hand, the resulting information could be used to give genetic counseling for pregnant women, such as early diagnosis of delayed deafness and drug-susceptible deafness. On the other hand, the information could be used to assess hearing conditions during pregnancy, leading to prevention and timely intervention for newborns.

## Author contributions

**Data curation:** Peng Zhou, Wei Lei, Yang Wang.

**Formal analysis:** Hua Hu, Jiayan Wu.

**Methodology:** Hua Hu.

**Supervision:** Ying Yang, Hailiang Liu.

**Visualization:** Hailiang Liu.

**Writing – original draft:** Wei Lei.

**Writing – review & editing:** Ying Yang.

## Supplementary Material

Supplemental Digital Content

## Supplementary Material

Supplemental Digital Content
